# Perception of Heaviness Induced by Sensorimotor Incongruence Is Associated with Pain Prognosis: A Pilot Study

**DOI:** 10.1155/2023/9906268

**Published:** 2023-04-04

**Authors:** Soichiro Matsuda, Michihiro Osumi

**Affiliations:** ^1^Graduate School of Health Science, Kio University, 4-2-2 Umaminaka, Kitakatsuragigun, Nara 635-0832, Japan; ^2^Neurorehabilitation Research Center, Kio University, Nara, Japan

## Abstract

**Background:**

Patients with chronic musculoskeletal pain experience not only pain but also abnormal body perception. Such abnormal body perception has been reported to be caused by incongruence between motor intentions and sensory feedback (i.e., sensorimotor incongruence). However, the influence of abnormal body perception with sensorimotor incongruence on pain prognosis in musculoskeletal pain patients has not been investigated.

**Objective:**

We aimed at clarifying the influence of abnormal body perception on pain prognosis using an experimental procedure for inducing sensorimotor incongruence in patients with musculoskeletal pain.

**Methods:**

We recruited 18 patients within 2 months after limb fracture or ligament injury. In the experiment, patients sat with the intact upper or lower limb reflected in a large mirror aligned with the sagittal plane. A motor task was performed for 20 seconds in each of the congruent and incongruent conditions. In the congruent condition, patients were asked to perform flexion-extension movements with the intact and affected limbs in-phase, while observing the intact limb in the mirror. In the incongruent condition, patients were asked to perform flexion-extension movements antiphase, while observing the intact limb in the mirror. After performing the congruent and incongruent conditions, patients were asked to complete a questionnaire about abnormal body perception. These procedures were conducted within 2 months after the fracture (first), 2 weeks after the first measurement (second), and 4 weeks (third) after the first measurement.

**Results:**

Pain, heaviness, and peculiarity were more likely to be experienced in incongruent conditions. Additionally, structural equation modeling indicated that heaviness at the first time point predicted the pain intensity at the second and third time points.

**Conclusions:**

Heaviness caused by sensorimotor incongruence may predict pain prognosis in patients with musculoskeletal pain after one month.

## 1. Introduction

Patients with chronic musculoskeletal pain suffer from not only pain but also abnormal body perception [1–8]. A previous study reported that cast immobilization immediately after a fracture or tissue injury caused abnormal body perception in addition to limiting physical function, such as joint contracture and muscle weakness [9]. This abnormal perception has been described by patients in clinical settings in various ways, such as “it does not feel like my hand,” and “I feel uncomfortable or strange about my hand” [10]. In several other studies, Patients with chronic musculoskeletal pain described their experience in the following ways: “I feel like my body is lead,” “I feel like my body is constricted,” “I feel like my body is swollen,” and “I feel discomfort in my body” [1, 8, 11, 12]. Such abnormal perception is thought to be caused by incongruence between motor intentions and sensory feedback [13–16]. In healthy subjects, the integration of motor intentions, output of motor commands, motor execution, and sensory feedback generate smooth human action. However, when there is an incongruence between the motor and sensory systems (i.e., sensorimotor incongruence), abnormal body perception is experienced [14, 16, 17]. For example, spatial incongruence between limb movements and visual feedback was reported to cause abnormal body perception (e.g., peculiarity, discomfort, heaviness, and pain) in healthy subjects [14]. Another study in healthy subjects reported that sensorimotor incongruence induced subjective experiences such as “heaviness,” “peculiarity,” and the perception of having an “extra limb” [17]. Interestingly, these abnormal body perceptions induced by sensorimotor incongruence are thought to be responsible for pathological pain. A previous systematic review of sensorimotor incongruence in musculoskeletal pain suggested that patients with musculoskeletal pain are more likely to experience abnormal perception because of sensorimotor incongruence, whereas sensorimotor incongruence does not directly cause pain [18]. For example, patients with whiplash injuries are more likely to experience “heaviness,” “peculiarity,” and “discomfort” than healthy subjects [19]. Another previous study reported that patients with complex regional pain syndrome and those with fibromyalgia are also more likely to experience abnormal body perception in a sensorimotor incongruence procedure compared with healthy subjects [15]. These previous findings suggest that abnormal body perception induced by sensorimotor incongruence may be closely related to chronic pain and could be an important factor in pain prognosis. In addition to clinical studies [18, 19], a basic study reported that abnormal perception with sensorimotor incongruence affected experimental pain in healthy subjects [20]. However, the effects of abnormal body perception with sensorimotor incongruence on pain prognosis have not been investigated. Considering these previous findings, we speculate that clinical acute pain may also be affected by abnormal perception. Therefore, we focused on the transition from acute to chronic pain and speculated that sensorimotor incongruence might be a factor in this transition. Therefore, a long-term evaluation of patients with musculoskeletal pain during the acute phase may be helpful for identifying differences in abnormal perception between patients with acute pain and those with chronic pain. This study confirmed the prognosis approximately 1 month after the first evaluation. In the current study, we examined which types of abnormal body perception are involved in pain prognosis using an experimental procedure for inducing sensorimotor incongruence. We hypothesized that the acute pain severity itself does not predict prolonged pain, but that abnormal perception may mediate it so that pain prognosis can be predicted. It seems that the study should be presented as a pilot study to determine sample sizes for future studies, since it is unclear if the study was appropriately powered.

## 2. Methods

### 2.1. Subjects

Sensorimotor incongruence has been suggested to affect not only the upper limbs but also the lower limbs [14]. Therefore, in this study, experiments were conducted with posttraumatic fracture patients of both upper and lower limbs. Patients were recruited within 2 months after upper and lower limb fracture and ligament injury (excluding fractures around the shoulder and hip joints). Patients with shoulder and hip joints fracture were not recruited because shoulder and hip joints cannot be reflected on the mirror then patients cannot see them and not experience sensorimotor incongruence.

The study was conducted at Setsunan General Hospital and patients were admitted to Setsunan General Hospital from April 2019 to April 2021. Patients were fully informed about the study and provided written consent. The study protocol conformed to the Declaration of Helsinki and it was approved by the Research Ethics Committee. Exclusion criteria were follows: impairment of the somatosensory processing or asymmetric limb disorders unrelated to the current injury (e.g. spinal cord injury, stroke) and dementia (23 < MMSE score).

### 2.2. Procedures

**Figure 1:**
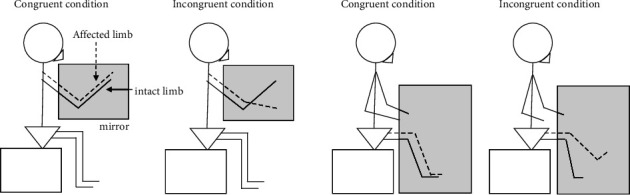
The patient was seated in a chair and a mirror (150 cm × 90 cm) was placed between the upper or lower extremities in the congruent and incongruent conditions. Congruent condition: the patient performed flexion-extension exercises on the healthy and affected sides at the same time. Incongruent condition: the patient performed flexion-extension exercises on the healthy and affected sides at different times. Each condition was performed for 20 seconds.

Before the experiment, patients completed the brief pain inventory (BPI) (pain intensity items only), [21] Pain Catastrophizing Scale (PCS)-6, [22] Tampa Scale for Kinesiophobia (TSK)-11, [23] central sensitization inventory (CSI)-9 [24], and Neglect-like Symptoms (NLS) questionnaire (7-point Likert scale from 0 to 6) [25]. The standardized pain questionnaires such as BPI, PCS, TSK, CSI, and NSL do not require copyright clearance. High PCS-6 score means patients are thinking catastrophic. High TSK-11 score means patients are feeling motor-related fear. High CSI-9 score means Central Sensitivity Syndrome. High NLS questionnaire score means somatic neglect symptoms for the painful site, such as the feeling that the painful area is not part of one's own body [26]. In the experiment, patients were seated in a chair, and a mirror (150 cm × 90 cm) was placed between the upper and lower limbs (Figure 1). In the experimental setup, patients could see their intact limb in the mirror, but could not see their affected limb [15]. The patients then performed the task in the congruent and incongruent conditions. The effect of sensorimotor incongruence is not explained to the patients, only the experimental procedures. In the congruent condition, patients were asked to perform flexion-extension movements with the intact and affected limbs in-phase. In the incongruent condition, patients were asked to perform flexion-extension movements with the intact and affected limbs antiphase. The congruent and incongruent conditions were performed for 20 seconds. After performing the task in the congruent and incongruent conditions, patients were asked to answer a questionnaire about abnormal body perception (pain, discomfort, peculiarity, heaviness, temperature change, extra limb, lost limb on a 7-point Likert scale (0 = Not at all and 6 = Very strong). These items of questionnaires were created based on previous study which analyzed abnormal body perception in patients with chronic low back pain [15]. The Abnormal Perception Questionnaire is consisted of pain (how much pain do you feel at your injured side), heaviness (How much heaviness do you feel at your injured side), temperature change (how much temperature change do you feel at your injured site), discomfort (how much discomfort do you feel at your injured side), peculiarity (how much peculiarity do you feel at your injured side), discomfort (how much discomfort do you feel at your injured site), extra limb (how much does your upper or lower limb increase sensation), and lost limb (how much does your upper or lower limb lost sensation). Then, to standardize the obtained data, Z-scores were then calculated by subtracting the population mean from each abnormal body perception value and dividing it by the standard deviation. The above evaluation was conducted within 2 months after the fracture (first time point), then twice more: 2 weeks after the first evaluation (second time point) and 4 weeks after the first evaluation (third time point). We extracted items from questionnaires with Z-scores of zero or greater, then used these items to represent experiences of abnormal perception. Patients were blinded to the experiment by not explaining the purpose of this study. Experimental data were blinded using assigning numbers so that data analysts were unable to identify individuals. It is possible to identify abnormal perceptions that are characteristic of an individual. For example, if a patient scores 5 points for heaviness and 2 points for other items, such as discomfort and temperature change, patient heaviness approaches 1 point, but the other items approach −1. Patients were blinded to the experiment by not explaining the purpose of this study. Experimental data were blinded using assigning numbers to that data analysts could not identify the individual. Patients were asked to report the average pain intensity from 24 h prior to the assessment, [15] so in this study, the average pain intensity was checked and those who had pain were included. We called such averaged pain “NRS (pain intensity).” In addition to that, we recorded pain intensity during sensorimotor incongruence procedure using mirror. We called such experimental pain “Pain (incongruent).”

### 2.3. Analysis

The results of the total pain scores on the NRS (0–10), PCS-6 (0–24), TSK-11 (11–44), CSI-9 (0–36), and NLS (0–30) questionnaires in each evaluation phase were compared using the Wilcoxon signed rank test. The abnormal body perception data in the congruent and incongruent conditions were not normally distributed in the Shapiro–Wilk test. Therefore, the Wilcoxon Signed rank test was used to compare body perception data between the congruent and incongruent conditions, and multiple comparisons were conducted at three evaluation time points: first, second and third time points. The level of significance was set at *p* < 0.05 for comparison between the congruent and incongruent conditions. The Bonferroni correction was used to adjust the *p* value obtained in the post hoc analysis for the comparison of the three evaluation time points. In this study, the significance level was set at *p* < 0.0167 because three comparisons were conducted across the three evaluation time points. Next, structural equation modeling (SEM) was used to examine the relationship between pain intensity and abnormal perception. Abnormal perception score with a *Z* score of 0 or more was used in SEM.

## 3. Results

There were 18 participants (tibial plateau fracture (*n* = 5), distal end of radius fracture (*n* = 5), distal end of femur fracture (*n* = 3), patella fracture [*n* = 1], trapezium fracture (*n* = 1), total knee replacement (*n* = 1), anterior cruciate ligament injury (*n* = 1), medial collateral ligament injury (*n* = 1)) in this study with a mean age of 70.7 ± 16.1 years. There were no interruptions to the study or missing data. The elapsed time from fracture or ligament injury to initial evaluation was 38.6 ± 11.8 days; the second and third evaluations were performed at 53.0 ± 12.9 days and 68.7 ± 14.1 days.

### 3.1. Clinical Questionnaires

The mean questionnaire scores at the first, second, and third evaluation time points, respectively, were as follows: the mean pain intensity scores were 2.5 ± 2.1, 1.7 ± 1.2, and 1.6 ± 1.5; the mean CSI-9 scores were 10.2 ± 4.0, 9.9 ± 5.2, and 8.3 ± 4.4; the mean PCS-6 scores were 9.8 ± 6.2, 7.6 ± 5.9, and 8.2 ± 5.5; the mean TSK-11 scores were 22.7 ± 4.7, 22.6 ± 6.1, and 20.6 ± 5.2; the mean TSK-11 scores were 22.7 ± 4.7, 22.6 ± 6.1, and 20.6 ± 5.2; and the mean NLS scores were 12.2 ± 6.6, 10.5 ± 6.4, and 8.1 ± 7.2. The comparison of the evaluation time points revealed no significant differences (Table 1).

### 3.2. Comparison of Congruent and Incongruent Conditions

**Figure 2:**
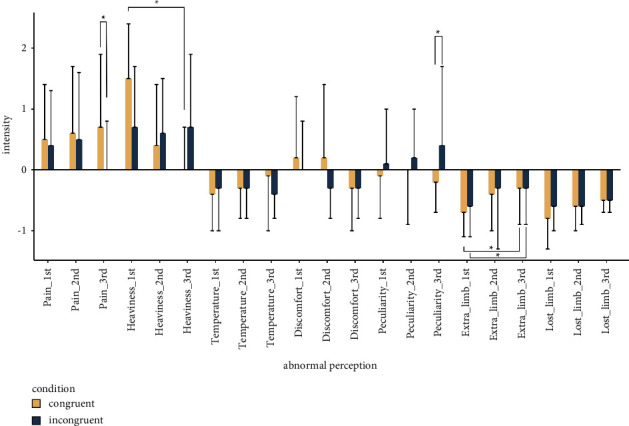
The intensity of standardized abnormal perception experienced in the congruent and incongruent conditions. In the congruent condition, there were significant differences between heaviness (first) and heaviness (second), and between heaviness (first) and heaviness (third). In the incongruent condition, there was a significant difference between extra limb (first) and extra limb (third). In the congruent condition and the incongruent condition, there was a significant difference in pain between the first and third time points.

The mean *Z*-scores ± SD of abnormal body perception (first, second, and third evaluation time points, respectively) in the congruent condition were as follows: pain, 0.5 ± 0.9, 0.6 ± 1.1, and 0.7 ± 1.2; heaviness, 1.5 ± 0.9, 0.4 ± 1.0, and 0.0 ± 0.7; temperature change, −0.4 ± −0.6, −0.3 ± −0.5, and −0.1 ± −0.9; discomfort, 0.2 ± 1.0, 0.2 ± 1.2, and −0.3 ± −0.7; peculiarity, −0.1 ± −0.7, 0.0 ± −0.9, and −0.2 ± −0.5; extra limb, −0.7 ± −0.4, −0.4 ± −0.6, and −0.3 ± −0.6; and lost limb, −0.8 ± −0.5, −0.6 ± −0.4, and −0.5 ± −0.2. In the incongruent condition, mean Z-scores ± SD of abnormal body perception (first, second, and third evaluation time points, respectively) were as follows: pain, 0.4 ± 0.9, 0.5 ± 1.1, and 0.0 ± 0.8; heaviness, 0.7 ± 1.0, 0.6 ± 0.9, and 0.7 ± 1.2; temperature change, −0.3 ± −0.7, −0.3 ± −0.5, and −0.4 ± −0.4; discomfort, 0.0 ± 0.8, −0.3 ± −0.5, and −0.3 ± −0.5; peculiarity, 0.1 ± 0.9, 0.2 ± 0.8, and 0.4 ± 1.3; extra limb, −0.6 ± −0.4, −0.3 ± −1.0, and −0.4 ± −0.6; and lost limb, −0.6 ± −0.4, −0.6 ± 0.3, and −0.5 ± −0.2. The results of multiple comparisons using Wilcoxon Signed rank test showed a significant decrease in heaviness and extra limb between the first time point and the third time point in the congruent condition (*p* < 0.01) (Figure 2). In the incongruent condition, the results of multiple comparisons revealed a significant decrease in lost limb between the first and third time points (*p* < 0.01) (Figure 2). In addition, there was a significant difference in pain and peculiarity at the third time point between the congruent and incongruent conditions (*p* < 0.01) (Figure 2).

### 3.3. Structural Equation Modeling (SEM)

**Figure 3:**
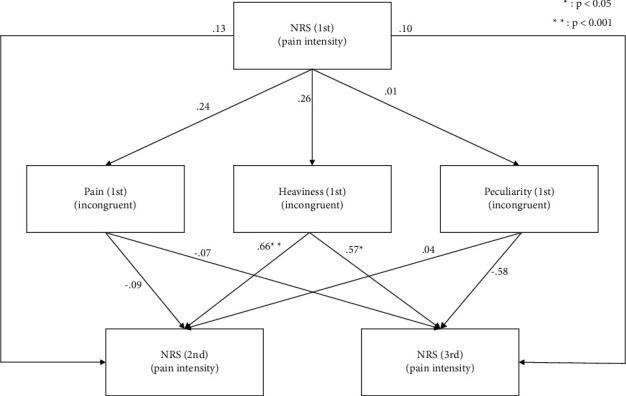
Structural equation model of pain intensity and abnormal perception. A significant positive correlation (*p* < 0.05) was found between the pain intensity (second and third) and heaviness (first).

On the basis of the *Z*-scored abnormal body perception in the incongruent condition, the items of pain, heaviness, and peculiarity were extracted, because subjects experienced abnormal body perception in the sensorimotor incongruence procedure (Figure 2). We then used these three items in the SEM procedure. The fit indices are shown in Table 2. The fit index values indicated that the model provided a good representation of the data. The standardized coefficients for each pass of the SEM and the plots of the extracted abnormal body perception and pain intensity are shown in Figure 3. The coefficients show a significant positive correlation between pain intensity and heaviness in the second or third time point, suggesting that heaviness in the first time point had a significant effect on subsequent pain intensity. The correlation between pain intensity and heaviness was confirmed in the post hoc analysis. The correlation between pain intensity (second and third) and heaviness (first) was calculated by Spearman's rank correlation coefficient. Statistical significance was set at *p* < 0.05.

Additionally, when the same items of abnormal body perception (i.e., pain, heaviness, and peculiarity) were used as variables in the congruent condition, the hypothesized model did not hold.

## 4. Discussion

The purpose of the current study was to examine abnormal body perception, which is related to pain prognosis in patients with acute musculoskeletal pain, using an experimental procedure for inducing sensorimotor incongruence. The results revealed that patients tended to experience pain, heaviness, and peculiarity as abnormal body perception in the incongruent condition. Importantly, SEM revealed that the intensity of pain at the first evaluation point did not predict the pain prognosis, whereas heaviness in the incongruent condition at the first evaluation time point may predict pain intensity at the second and third evaluation time points.

Patients experienced pain, heaviness, and peculiarity in experimental situations in which the motor intention and sensory feedback were incongruent. Previous studies reported that pain sensation and peculiarity can be induced by experimental conditions involving the presentation of visual feedback that is opposite to the direction of body movement [14, 15, 18, 27]. The sensation of heaviness was reported in an experimental condition in which visual feedback was given with a time delay [28]. Additionally, regarding the sensation of heaviness, the size-weight illusion is a well-known phenomenon [29]. In this illusion, participants over-estimate the weight of a smaller object compared with a larger object of the same mass [29]. Pain, heaviness, and peculiarity are all caused by sensorimotor incongruence. For example, when visual feedback that is opposite to the direction of body movement is presented, motor intention and sensory feedback are not congruent. Considering such sensorimotor incongruence causes abnormal sensation, our current results suggest that experimental sensorimotor incongruence caused pain, heaviness, and peculiarity in patients with musculoskeletal pain may not be surprising.

SEM results revealed that pain intensity at the first evaluation time point did not predict pain prognosis, but heaviness in the incongruent conditions at the first evaluation time point may predictive of pain prognosis after one month. Many clinical outcomes have been used to predict pain prognosis [30, 31]. Abnormal body perception is also known as a factor influencing pain prognosis. For example, distorted body image, such as feeling as though one's arms are swollen, has been reported to be associated with chronic pain [32, 33]. However, a previous study assessed body perception based on patient-reported outcomes (PROs) [34–36]. Although PROs are useful in clinical settings, patients are sometimes not aware of their abnormal body perception. In this case, outcome scores are classified as “not distorted body perception” although patients implicitly exhibit abnormal body perception. In contrast, experimental procedures for inducing sensorimotor incongruence, such as the method used in the present study, can induce abnormal body perception and make patients aware of their abnormal body perceptions. In clinical settings, a sensorimotor incongruence procedure could be useful for assessing abnormal body perception in musculoskeletal pain patients for prediction of pain prognosis after one month.

Considering the findings described above, we present a possible rehabilitation approach using mirror visual feedback (so-called “mirror therapy”) below, on the basis of our results. Mirror therapy has been used to match motor intentions and visual feedback, and then alleviate pain. Although many previous studies have been conducted in patients with chronic pain [37–42], some patients do not respond well to mirror therapy [43]. For example, some patients experience aggravation of pain and dizziness [44]. It is unclear why some patients do not exhibit an improvement of pain with mirror therapy. We propose that this phenomenon may indicate a problem with the details of the mirror therapy procedure. It is possible that patients who do not benefit from mirror therapy may be exposed to sensorimotor incongruence between the immobile affected limb and the smoothly moving intact limb in the mirror during mirror therapy, resulting in abnormal body perception. This abnormal body perception might interrupt pain alleviation. Thus, a slight sensorimotor incongruence in mirror therapy may interfere with pain alleviation.

The present study involved several limitations. First, there was no significant difference between abnormal perception in the congruent and incongruent conditions, except for pain and peculiarity at the third evaluation time point, in which pain was more likely to be experienced in the incongruent compared with the congruent condition. These results might depend on muscle weakness in patients in the acute or subacute phase. Thus, there was a slight incongruence between the left and right upper or lower limbs because of muscle weakness in the affected limb. A previous study reported that a slight delay of visual feedback (e.g., 250 ms delay) can cause abnormal body perception [27]. Considering this finding, the perception of heaviness in the congruent condition may indicate that muscle weakness on the affected side influenced the results, regardless of the presence or absence of pain. In future, the procedure for inducing sensorimotor incongruence should be performed with a defined motor speed and rhythm to avoid slight spatial incongruence. Imai et al. reported pain trajectory can predict the pain prognosis such as distal radius fracture [45, 46]. Additionally, it is known that not only pain intensity but also anxiety and catastrophizing are involved in acute pain prognosis [30]. In addition, pain intensity and swelling have been found to be predictors of the development of CRPS after traumatic upper limb fracture [47, 48]. This study suggests that not only these physical characteristics but also the psychological experience of sensorimotor incongruence may influence the pain prognosis after one month. In this study, patients who experienced heaviness were more likely to have prolonged pain. In the future, it will be possible to determine the cutoff of abnormal perception experienced by patients with prolonged pain to improve the accuracy of prognosis prediction in clinical practice. Importantly, SEM revealed that the intensity of pain at the first evaluation point did not predict the pain prognosis, whereas heaviness in the incongruent condition at the first evaluation time point predicted pain intensity at the second and third evaluation time points. Therefore, in clinical practice, attention should be paid not only to the pain intensity but also to patients' subjective experience with the sensorimotor incongruence procedure using a mirror. If patients experience subjective heaviness with the incongruence condition, clinicians predict the patient's prognosis worth and should add other treatment. Thus, for estimating pain prognosis after one month, it is more important to evaluate abnormal perception in the incongruent condition than in the congruent condition.

In future, it will be possible to determine the cutoff of abnormal perception experienced by patients with prolonged pain to improve the accuracy of prognosis prediction in clinical practice. Although a small sample size is sufficient if the model is simple, a sample size of 100 or more is recommended [49]. This study was conducted with 18 subjects as a pilot study. In the future, we will collect data from more than 100 patients to investigate the effects of abnormal perception on pain. On the other hand, almost patients in the present study had moderate pain but did not suffer from severe pain. It is unclear in the present study whether the prognosis for patients with musculoskeletal pain with low pain intensity was similar to that of patients with high pain intensity. In the future, we plan to investigate the effect of abnormal perception on the prognosis of patients suffering from high pain intensity. Patients with high pain intensity are known to have a poor prognosis. However, in the current study, the number of patients with high pain intensity was small. Therefore, the factor of heaviness experience would be remarkable rather than pain intensity in the present study. We intend to investigate the effect of abnormal perception for high pain intensity patients on the pain prognosis as we continue to add to our data.

## 5. Conclusion

Heaviness induced by sensorimotor incongruence suggests that it may be predictive of pain prognosis after one month in patients with acute musculoskeletal pain. However, data needs to be collected further because the patients have pain with low intensity and the number of patients is small. Thus, we do not know the long-term prognosis because we have not yet completed the long-term follow-up. This is a matter for further study. As more data become accumulated, the influence of abnormal perception on the pain prognosis will become clearer.

## Figures and Tables

**Table 1:** Comparison of the time points of each evaluation did not reveal any significant differences.

	1^st^	2^nd^	3^rd^
Time from fracture (days)	38.6 ± 11.8	53.0 ± 12.9	68.7 ± 14.1
NRS (pain intensity) (0–10)	2.5 ± 2.1	1.7 ± 1.2	1.6 ± 1.5
CSI-9 (0–36)	10.2 ± 4.0	9.9 ± 5.2	8.3 ± 4.4
PCS-6 (0–24)	9.8 ± 6.2	7.6 ± 5.9	8.2 ± 5.5
TSK-11 (11–44)	22.7 ± 4.7	22.6 ± 6.1	20.6 ± 5.2
NLS (0–30)	12.2 ± 6.6	10.5 ± 6.4	8.1 ± 7.2

CSI-9; central sensitization inventory-9, PCS-6; Pain Catastrophizing Scale-6, TSK-11; Tampa Scale for Kinesiophobia-11, and NLS; neglect-like symptoms.

**Table 2:** The fit index values indicated that the model provided a good representation of the data.

	Χ^2^/df	GFI	AGFI	RMSEA
Proposed model	0.605	0.986	0.903	0.000
Standard of good fit	<2.0	0.90<	0.90<	<0.08

GFI: goodness of fit index; AGFI: adjusted goodness of fit index; and RMSEA: root mean square error of approximation.

## Data Availability

The data used in this study are available upon reasonable request from the corresponding author.
